# Short-Term Soy Isoflavone Intervention in Patients with Localized Prostate Cancer: A Randomized, Double-Blind, Placebo-Controlled Trial

**DOI:** 10.1371/journal.pone.0068331

**Published:** 2013-07-12

**Authors:** Jill M. Hamilton-Reeves, Snigdha Banerjee, Sushanta K. Banerjee, Jeffrey M. Holzbeierlein, J. Brantley Thrasher, Suman Kambhampati, John Keighley, Peter Van Veldhuizen

**Affiliations:** 1 Department of Dietetics and Nutrition, University of Kansas Medical Center, Kansas City, Kansas, United States of America; 2 Division of Hematology and Oncology, Department of Medicine, University of Kansas Medical Center, Kansas City, Kansas, United States of America; 3 Department of Anatomy and Cell Biology, University of Kansas Medical Center, Kansas City, Kansas, United States of America; 4 Department of Urology, University of Kansas Medical Center, Kansas City, Kansas, United States of America; 5 Department of Biostatistics, University of Kansas Medical Center, Kansas City, Kansas, United States of America; 6 Cancer Research Unit, V.A. Medical Center, Kansas City, Missouri, United States of America; Wayne State University School of Medicine, United States of America

## Abstract

**Purpose:**

We describe the effects of soy isoflavone consumption on prostate specific antigen (PSA), hormone levels, total cholesterol, and apoptosis in men with localized prostate cancer.

**Methodology/Principal Findings:**

We conducted a double-blinded, randomized, placebo-controlled trial to examine the effect of soy isoflavone capsules (80 mg/d of total isoflavones, 51 mg/d aglucon units) on serum and tissue biomarkers in patients with localized prostate cancer. Eighty-six men were randomized to treatment with isoflavones (n = 42) or placebo (n = 44) for up to six weeks prior to scheduled prostatectomy. We performed microarray analysis using a targeted cell cycle regulation and apoptosis gene chip (GEArrayTM). Changes in serum total testosterone, free testosterone, total estrogen, estradiol, PSA, and total cholesterol were analyzed at baseline, mid-point, and at the time of radical prostatectomy. In this preliminary analysis, 12 genes involved in cell cycle control and 9 genes involved in apoptosis were down-regulated in the treatment tumor tissues versus the placebo control. Changes in serum total testosterone, free testosterone, total estrogen, estradiol, PSA, and total cholesterol in the isoflavone-treated group compared to men receiving placebo were not statistically significant.

**Conclusions/Significance:**

These data suggest that short-term intake of soy isoflavones did not affect serum hormone levels, total cholesterol, or PSA.

**Trial Registration:**

ClinicalTrials.gov NCT00255125

## Introduction

Prostate cancer is the most common non-cutaneous cancer in American men [1]. Epidemiological studies associate soy food consumption in Asian populations with a decrease in prostate cancer risk [2], [3], although plasma genistein concentration has not been associated with prostate cancer risk at dietary exposures typical of Western populations [2]–[4]. The circulating soy isoflavone concentrations in Japanese men are at least 10-fold higher than American [5], [6] and European men [4]. A typical soy-rich Japanese diet consists of 25–100 mg soy isoflavones/day, while a typical American diet contains about 2–3 mg soy isoflavones/day [7]. Migration studies of Asian men suggest that their prostate cancer incidence rates increase in direct relation to the length of time spent residing in the United States [8], [9]. Likewise, prostate cancer incidence and mortality has increased in Asian countries concurrently with the Westernization of their diet [10].

The components of the soy diet thought to suppress cancer are the soy isoflavones daidzein, genistein, and glycitein. Soy isoflavones bind to estrogen receptors and exert hormonal effects. The two primary isoflavones in soybeans are genistein and daidzein. Daidzein is converted to equol in the gut of certain individuals. Isoflavones accumulate in the prostate gland [10] and have been shown to modulate endogenous hormones relevant to prostate carcinogenesis [11], [12]. Soy isoflavones also exert nonhormonal effects to suppress cancer through pathways that target cell cycle and apoptosis such as G2/M arrest and p21 expression *in vitro* in androgen independent PC3 prostate cancer cells [13]. Soy isoflavone induced inhibition of cell proliferation and induction of apoptosis are partly mediated through the regulation of the Akt/FOXO3a/GSK-3beta/AR signaling network [14].

Short term soy isoflavone interventions in patients with prostate cancer have yielded mixed results. In a nonrandomized, non-blinded trial of 38 patients (20 with clinically significant prostate cancer and 18 controls), a daily intake of 160 mg/d of isoflavones derived from red clover for about 20 days until radical prostatectomy led to significantly higher apoptosis of tumor cells [15]. In men with prostate cancer, soy or isoflavone consumption has significantly decreased mean total serum PSA compared to controls [16]–[18], although several studies have not shown statistically significant effects on PSA in this population [15], [19], [20]. The cholesterol-lowering effects of soy foods are primarily thought to be mediated through the soy protein [21]; although recently, a genistein-only intervention in patients with localized prostate cancer significantly decreased total cholesterol and this change was statistically significant as compared to placebo [18].

We have already published data from a pilot trial conducted by our laboratory in a cohort of 13 prostate cancer patients (without any prior therapy) who were treated with a soy isoflavone supplement containing 112 mg total isoflavones (n = 4), 168 mg total isoflavones (n = 3), or 224 mg total isoflavones (n = 4). These pilot data showed a reduction in PSA, testosterone, and estrogen, and a variable effect on ERα expression with down-regulation of receptor expression seen at the 224 mg total isoflavones per day dose level while there was no observed effect on AR expression [22].

The present double-blinded, randomized, placebo-controlled trial was designed to more fully investigate the effectiveness of total soy isoflavone consumption in a larger cohort of men by monitoring serum hormone levels, total cholesterol, and PSA. In addition, we evaluated the effects on tumor and adjacent normal prostate tissue with gene arrays for the regulation of cell-cycle control and apoptosis in men with localized prostate cancer.

## Results

### Study Details

**Figure 1:**
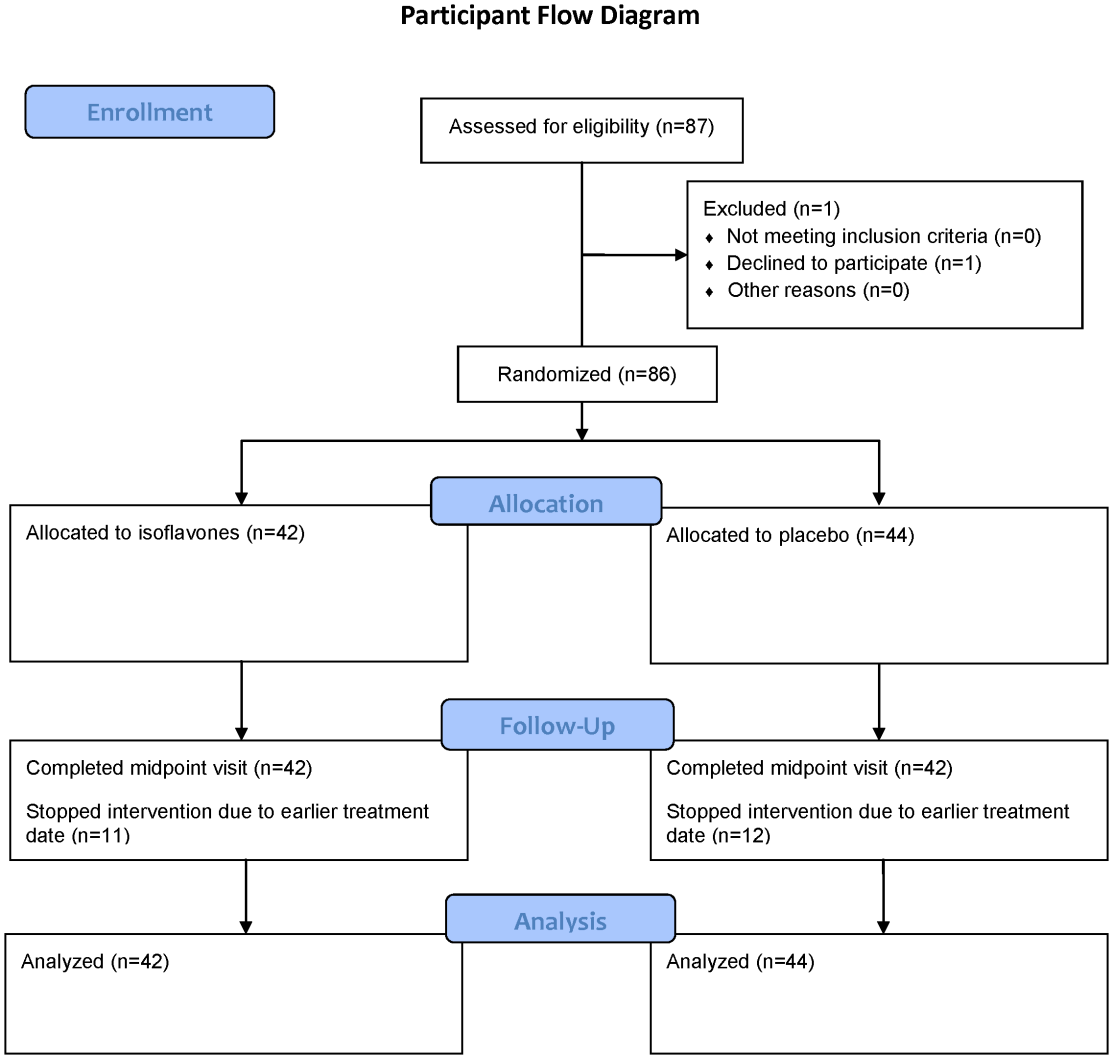
Participant Flow Diagram.

Eighty-seven subjects were screened for the study; 1 chose not to participate (FIGURE 1). Twenty-three subjects did not complete the third visit because their surgery or radiation therapy treatment date occurred before the last visit was scheduled. Fifty-six of the patients underwent prostatectomy [placebo (n = 27), soy isoflavone (n = 29)]. Twenty-eight subjects elected radiation therapy at study end [placebo (n = 16), soy isoflavone (n = 12)] and 2 subjects chose active surveillance [placebo (n = 1), soy isoflavone (n = 1)]. All 86 subjects completed 2 weeks of the study with good compliance, and 63 subjects completed the full study. Of the 86 participants, 74 urine samples from the 2-week time point were analyzable for urinary phytoestrogen metabolites (daidzein, genistein, glyctein, O-desmethylangolensin, dihydrodaidzein, and equol). Participants agreed to avoid dietary soy intake and not to take any new supplements. However, two participants in the placebo group showed high concentrations of urinary phytoestrogen metabolites, suggesting they were consuming soy from other sources. All participants analyzed in the intervention group appeared to be in compliance, and 29% (10/35) were classified as equol producers by determining if their log 10 (equol:daidzein) ratio was greater than −1.75 [23].

### Adverse Events

All adverse events were Grade 1 (mild). In the isoflavone arm, 4 events were recorded: 2 gastrointestinal and 2 general. In the placebo arm, 9 events were recorded: 6 gastrointestinal and 3 general. No patient stopped treatment because of adverse events.

### Baseline Characteristics

Baseline anthropometrics, demographics, clinical stage, and physical activity did not differ among the groups (TABLE 1). There were no differences in baseline serum hormone, cholesterol, and PSA concentrations between the groups (TABLE 2).

**Table 1:** Baseline Characteristics of Participants.

Characteristic	Isoflavone Group n = 42 men	Placebo Group n = 44 men
Mean age, y (sd)	62±12	62±7
Race, No. (%)		
White	38 (90)	31 (70)
Black	3 (7)	9 (20)
American Indian or Alaska Native	1(2)	4 (9)
Asian/Pacific Islander	0	0
Ethnicity, No. (%)		
Hispanic	1 (2)	2 (5)
Non-Hispanic	41 (98)	42 (95)
Clinical Stage, No. (%)^1^		
T1	15 (35)	24 (55)
T2	27 (64)	19 (43)
Mean baseline PSA, ng/mL (SD)	9±5	8±4
Gleason Score, No. (SD)	7±0.9	7±0.7
Body weight, pds (SD)	208±40	213±41
Height, in (SD)	70±2	70±3
BMI, kg/m^2^ (SD)	30±5	31±5
Physical Activity		
≥15 min exercise/day, No. (%)	8 (19)	6 (14)
<15 min exercise/day, No. (%)	34 (81)	37 (86)

Baseline characteristics were compared using t-tests for continuous variables after checking normality assumptions and Fisher’s Exact test for categorical variables. P<0.05 was considered statistically significant. PSA = prostate-specific antigen; BMI = Body Mass Index.

1 Clinical stage based on the American Joint Committee on Cancer criteria.

**Table 2:** Prostate Specific Antigen, Serum hormones, and Total Cholesterol in men with prostate cancer who consumed isoflavones or placebo for up to 6 weeks.

Serum marker		Isoflavone Group Mean ± SD, (n)	Placebo Group Mean ± SD, (n)
Prostate Specific Antigen, ng/mL			
Baseline	9±5 (42)		8±4 (44)
1 wk	9±3 (5)		6±6 (5)
2 wk	8±5 (31)		8±4 (37)
3 wk	8±5 (14)		5±3 (8)
4 wk	8±5 (9)		7±5 (6)
5 wk	6±4 (2)		10±4(5)
6 wk	7±3 (4)		10±4 (3)
Total Testosterone, ng/dL			
Baseline	345±135 (34)		363±150 (37)
1 wk	367±216 (4)		288±170 (4)
2 wk	376±152 (26)		405±173 (34)
3 wk	351±96 (11)		376±192 (6)
4 wk	368±142 (9)		497±270 (5)
5 wk	418±0 (1)		335±44 (5)
6 wk	231±51 (4)		276±98 (3)
Free Testosterone, pg/mL			
Baseline	48±24 (41)		43±22 (42)
1 wk	47±32 (5)		40±30 (5)
2 wk	56±16 (30)		51±23 (37)
3 wk	42±24 (13)		47±33 (8)
4 wk	38±24 (9)		73±48 (6)
5 wk	24±33 (2)		46±9 (5)
6 wk	51±2 (4)		44±6 (3)
Total Estrogen, pg/mL			
Baseline	130±84 (42)		136±76 (44)
1 wk	93±14 (5)		126±63 (5)
2 wk	130±58 (31)		131±56 (36)
3 wk	117±72 (14)		126±67 (8)
4 wk	107±48 (8)		133±17 (4)
5 wk	64±29 (2)		113±24 (5)
6 wk	177±91 (4)		149±83 (3)
Estradiol, ng/mL			
Baseline	20±13 (39)		22±14 (40)
1 wk	22±14 (5)		21±10 (5)
2 wk	24±15 (36)		18±12 (36)
3 wk	24±9 (8)		16±14 (8)
4 wk	29±23 (5)		28±17 (5)
5 wk	36±25 (3)		30±14 (3)
6 wk	11±10 (2)		23±6 (2)
Total Cholesterol, mg/dL			
Baseline	177±38 (40)		186±39 (41)
1 wk	184±45 (5)		199±36 (4)
2 wk	167±34 (30)		180±35 (36)
3 wk	161±48 (12)		175±54 (7)
4 wk	201±46 (8)		187±38 (6)
5 wk	165±11 (2)		179±18 (5)
6 wk	168±39 (4)		155±13 (3)

Data are means+SD. Analysis of covariance model was used to compare groups adjusted for the baseline value of the outcome. *P*<0.05 was considered statistically significant.

Every patient had a maximum of 3 observations for each endpoint but the time of observation varied across patients due to the varied scheduling of the patients’ prostate cancer treatments.

### Serum Markers

No significant modulations of serum total testosterone, free testosterone, total estrogen, estradiol, prostate specific antigen, or total cholesterol were observed in the isoflavone-treated group compared to men receiving placebo (TABLE 2).

### Tissue Markers

#### Cell cycle gene expression

**Figure 2:**
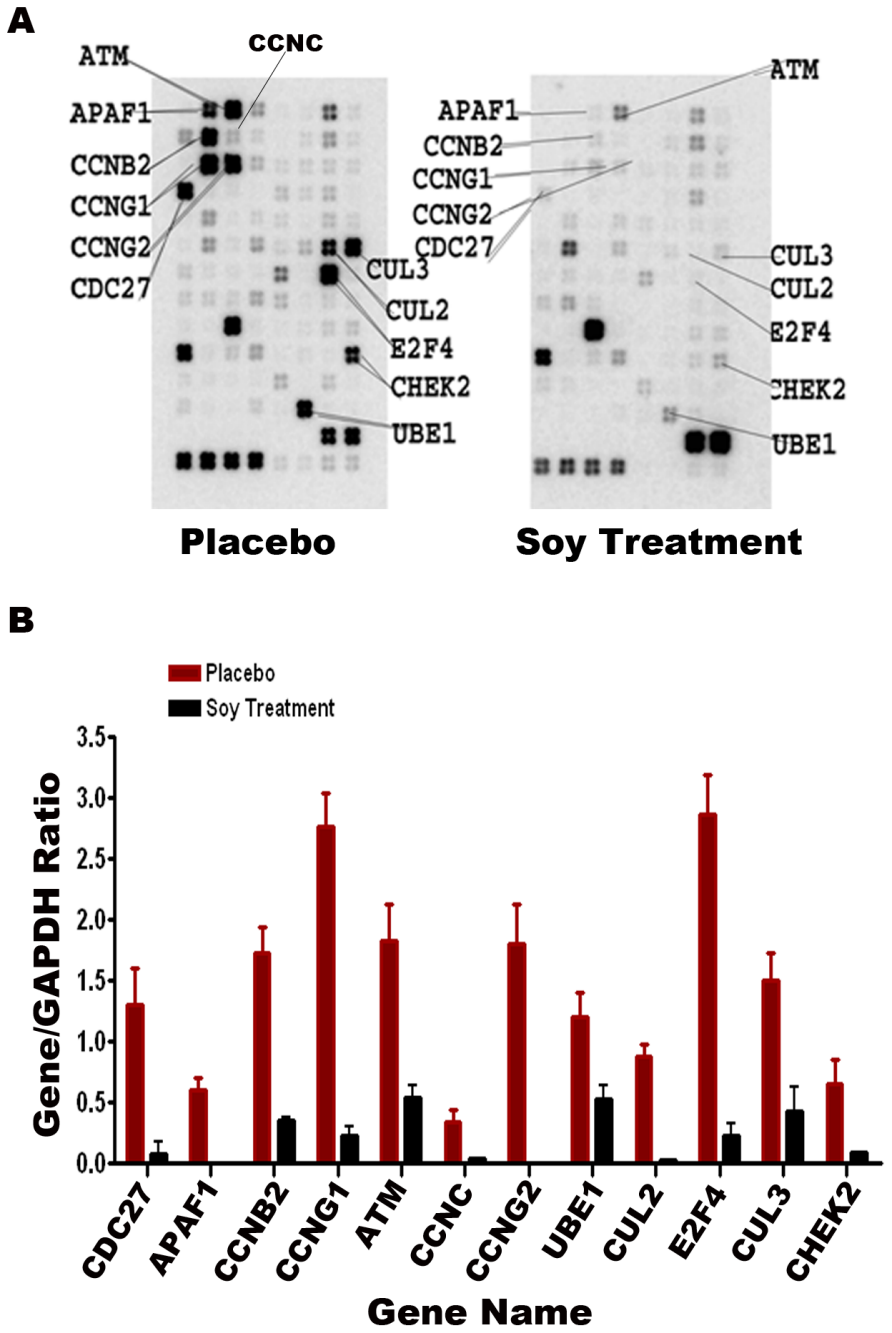
Cell cycle gene array of the prostate tumor specimen obtained from two patients with prostate cancer, one who consumed isoflavones and one who took the placebo. The figure shows the ratio of the gene to GAPDH.

Twelve genes in involved in the cell cycle were down-regulated in soy-treated malignant prostate tissue when compared to placebo treated malignant tissue, respectively (FIGURE 2). The cell cycle gene array demonstrated down-regulation of cell division cycle 27 (CDC27), involved in protein-protein interactions and timing of mitosis; apoptotic peptidase activating factor 1 (APAF1), involved in initiating apoptosis; cyclin B2 (CCNB2), involved in transforming growth factor beta-mediated cell cycle control; cyclin G1 (CCNG1) and cyclin G2 (CCNG2), activated by tumor protein p53; cyclin C (CCNC), involved in phosphorylation of RNA polymerase II; ubiquitin-like modifier activating enzyme 1 (UBE1), involved in marking cellular proteins for degradation; cullin 2 (CUL2) and cullin 3 (CUL3); E2F transcription factor 4 (E2F4); checkpoint kinase 2 (CHEK2) gene expression; and ataxia telangiectasia mutated (ATM), an important cell cycle checkpoint.

#### Apoptosis gene expression

**Figure 3:**
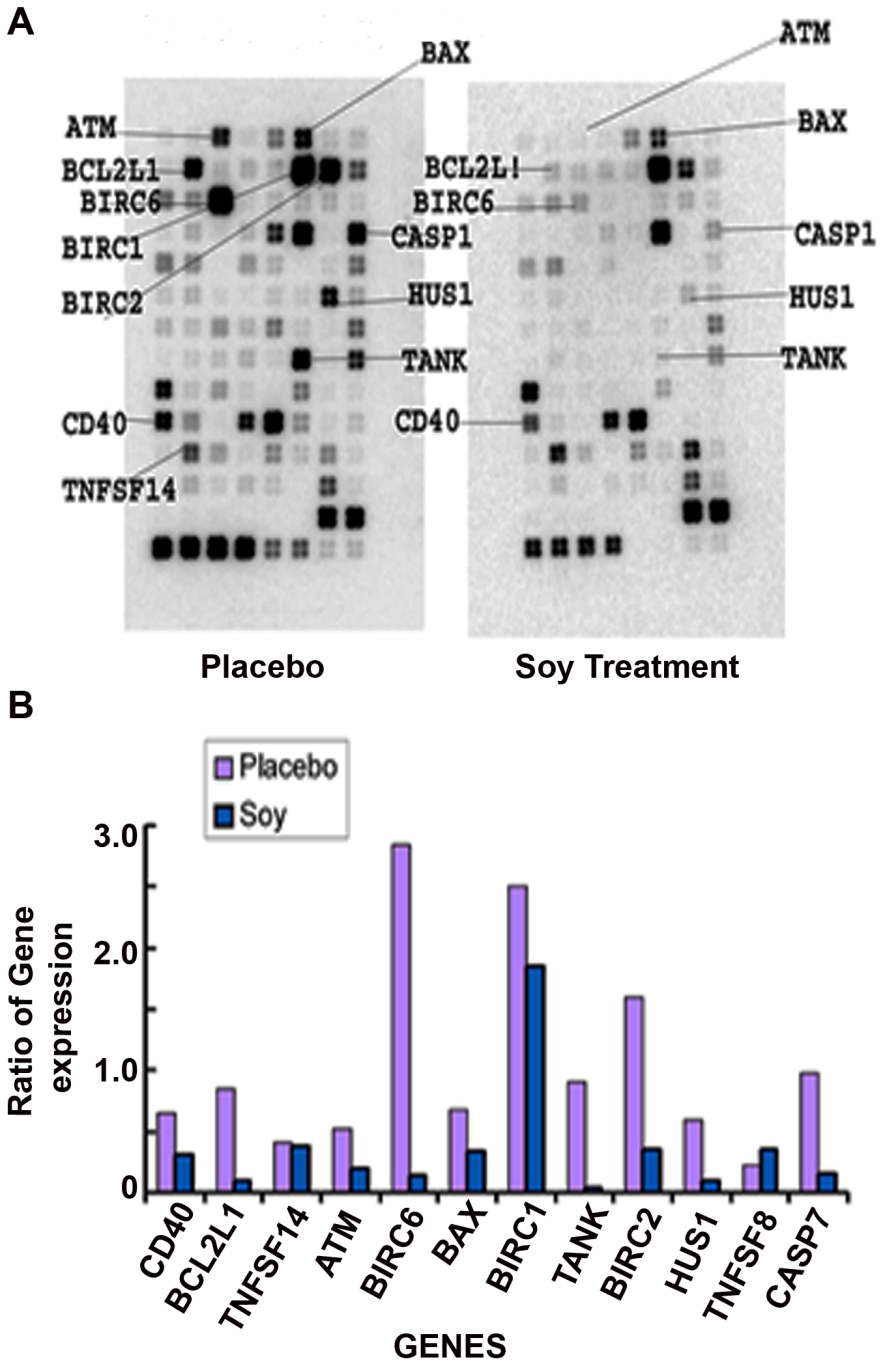
Apoptosis gene array of the prostate tumor specimen obtained from two patients with prostate cancer, one who consumed isoflavones and one who took the placebo. The figure shows the ratio of the gene to GAPDH.

Nine genes in involved in apoptosis were down-regulated in soy-treated malignant prostate tissue when compared to placebo treated malignant tissue, respectively (FIGURE 3). The apoptosis gene array demonstrated down-regulation of CD40, involved in a variety of immune and inflammatory responses; B-cell non-Hodgkin lymphoma-2 (Bcl-2); baculoviral IAP repeat containing 1, 2, and 6 (BIRC1/NAIP, BIRC2, BIRC6), involved in blocking apoptosis; BCL2-associated X protein (BAX); TRAF family member-associated NFKB activator (TANK); HUS1, and caspase 7 (CASP7) gene expression.

## Discussion

Despite data from our pilot investigation [22], we did not observe significant changes in PSA or hormone concentrations in this larger phase II clinical trial. Our earlier study reported a reduction in PSA, testosterone, and estrogen in 13 patients consuming the same isoflavone supplement at various dosages. Although the supplement formulation was identical in both studies, the differences in outcome may be due to the larger sample size and the wide variability of PSA and serum hormones in the present study. Our exploratory analysis in the current study suggests that the intake of soy isoflavones may alter gene expression in prostate tissue.

Our results for serum hormones are consistent with previous studies. A meta-analysis of 32 studies published in 2010 by Hamilton-Reeves et al. reported that consumption of soy foods is not associated with significant effects on circulating testosterone or free testosterone in men [24]. Recent publications since the meta-analysis have not shown significant effects of dietary intake of isoflavones on testosterone [18], [25], [26]. Data for blood concentrations of estrogen are available for fourteen studies, consisting of five randomized controlled trials [11], [27]–[30]. Only one study reported a significant increase in blood estradiol concentrations from baseline with 40 mg/d of isoflavones but not 60 or 80 mg/d of isoflavone supplementation, and statistical comparison to the placebo group was not published [29]. In summary, total isoflavone intake does not appear to influence circulating sex hormones at intakes comparable to dietary intake in Asian countries like the dose administered in the present study.

Our findings regarding total cholesterol are in contrast to a meta-analysis showing a decrease of total cholesterol with soy isoflavones when consumed in soy foods [21]. The theory that components from the soy protein itself lower cholesterol could explain why isoflavones only would not elicit the same effect. However, a recent report showed a 4% decrease in total cholesterol in prostate cancer patients who consumed 30 mg of synthetic genistein compared to placebo [18]. The likely explanation for these contrasting results compared to our study is due to the supplement formulation given in this trial. The soy isoflavone supplement was derived from soy germ which provides a mix of soy isoflavones that is higher in daidzein than genistein. Furthermore, we did not stratify our randomization based on the pre-treatment values of total cholesterol, although we did include baseline value as a covariate in the statistical model.

Isoflavone supplementations at doses similar to this study have generally shown no effect on lowering PSA concentrations in men with localized prostate cancer. The results for serum PSA are consistent with some [19], [20], [28], [31], but not all reports [16], [17], [32]–[34]. Total PSA reduced by 4% when an isoflavone-rich supplement was given as part of an intensive diet and lifestyle intervention in prostate cancer patients which suggests more dramatic changes in dietary intake than adding isoflavones may be needed to alter PSA in men with localized prostate cancer [16]. It is possible that a longer intervention or follow-up may have yielded a significant inhibitory effect of isoflavones on PSA, only 2 cases from week 5 and 4 cases from week 6 (out of 42 cases from the isoflavone treatment group) were available for PSA testing.

To our knowledge, this study was one of the first to compare the effects of total soy isoflavones consumption on gene expression in the prostate tissue obtained from a human intervention study. However, there are several limitations of our study including lack of stratification of results based on Gleason score, pathologic stage, or PSA. This may influence the effect of soy particularly on apoptotic pathways. In addition, only a very small number of tissue samples were analyzed, thus the results must be validated with a larger sample. Furthermore, given the multiple foci of cancer, the gene array differences from the samples between groups may not represent the gene expression in the tissue as a whole. Again, the small sample size may have led to sampling bias; however, we evaluated both tumor and normal tissue and would predict that the effect of soy isoflavones effect would be equivalent throughout the tissue.

### Conclusions

Intake of soy isoflavones appear safe with only mild adverse events reported and no effects on serum hormones. Further evaluation of the role of soy isoflavones in inducing apoptosis and cell cycle control is warranted in the preventive and therapeutic setting.

## Materials and Methods

### Ethics Statement

The KUH Institutional Review Board: Human Subjects Committee and the Kansas City VAMC Institutional Review Board approved the study protocol. All patients signed an informed consent prior to study enrollment. The protocol for this trial and supporting CONSORT checklist are available as supporting information; see Checklist S1 and Protocol S1.

### Patients

Between April 2006 and May 2009, 86 men with localized prostate cancer were recruited from the University of Kansas Hospital (KUH) Urology Clinic and the Kansas City Veterans Affairs Medical Center (VAMC). Patients with a clinical stage T1 or T2 prostate cancer (utilizing American Joint Committee on Cancer criteria) and were deemed surgical candidates were eligible for the study. Patients on concurrent chemotherapy, radiation or neoadjuvant hormone therapy were excluded from the study. Patients who consumed soy foods within 90 days prior to enrollment were also excluded, and prior soy intake was determined at baseline by using the validated soy foods questionnaire [35]. Patients with a known history of soy allergy or intolerance were also excluded. During the course of the study, patients agreed to avoid dietary soy intake and not to take any new vitamin supplementations or herbal remedies.

### Study Design

A double-blinded, randomized, placebo-controlled parallel trial was conducted, in which 86 men with a histologic diagnosis of prostate cancer who were scheduled to undergo prostatectomy received either soy isoflavone capsules (Flav-ein, 3 B’S Ltd, Lenexa, KS) or matching placebo capsules. Patients were sequentially randomized into one of the two arms. Random permuted block randomization was utilized to keep the allocations balanced at various points over the course of patient enrollment. The statistician generated codes and provided a copy of them to the pharmacists. The pharmacy distributed the supplements to the patients. The blind was maintained by the pharmacist and the statistician until the end of the study. The 42 patients randomized to soy isoflavones received capsules contributing 51 mg total isoflavone aglycone equivalents/d with a mean distribution of isoflavones of 55% daidzein, 30% glycitein, and 15% genistein as analyzed by NP Analytical Laboratories in St. Louis, MO. The 44 patients receiving placebo supplementation consumed <0.06 mg total isoflavones aglycone equivalents/d. Both groups consumed the capsules in divided doses twice daily. The patients took their last dose of the supplement the night prior to admission for surgery. The capsules were purchased in bulk in order to minimize variation in isoflavone content that can occur from batch to batch. Subjects were instructed to consume their habitual diets, and to exclude soy products in order to minimize isoflavone consumption from other sources. To assess compliance at the 2-week time point, the urinary concentration of six isoflavonoids [genistein, daidzein, glycitein, equol, DHD and ODMA] were measured at the University of Minnesota using a high pressure liquid chromatography-tandem mass spectrometry (LC-MS/MS) method modified from published methodology [36]. Data were normalized by adjusting with creatinine excretion and urinary creatinine was analyzed by the Kansas City VA Medical Center using a kinetic alkaline picrate method (Abbott 8200, Abbot Laboratories., Abbott Park, IL).

### Serum Collection and Analysis

Fasting blood was collected in the morning at 0, 2 weeks, and the day before the scheduled surgery or curative treatment. The last time point varied by subject based on the scheduled date for surgery. Treatment was never delayed to meet the study requirements. Serum was separated and sent to Quest Diagnostics (Lenexa, KS) for analysis. Serum samples were analyzed for total testosterone by liquid chromatography with tandem mass spectrometry, free testosterone by equilibrium dialysis, total estrogen by radioimmunoassay, estradiol and prostate specific antigen (PSA) by chemiluminescence immunoassay, and total cholesterol by enzymatic assay.

### Tissue Collection and Analysis

At the time of surgery and while still in the operating suite, the urological surgeon obtained core samples of both the adjacent normal prostate and tumor tissue from each prostate specimen. In brief, the prostate was sectioned and the tumor identified based on palpation, visualization and known location of the cancer. These cores were then placed immediately in liquid nitrogen and transferred to the laboratory for processing. In the laboratory a thin (5 µm) frozen section of the core was obtained and H&E stained. This section was then reviewed by a pathologist who confirmed the presence of cancer or normal tissue in each core specimen. The core sample was then labeled, placed in storage media, and maintained until analysis. This core collection process did not interfere with the subsequent pathologic processing and tumor assessment.

### Cell Cycle and Apoptosis Gene Arrays

In order to identify the soy-dependent cell cycle and apoptosis related gene expression profile in tumors samples, we used human cell cycle and apoptosis SuperArray gene expression analysis kits (GEArray-Q series 01001, Super Array Co, MD). Total RNA was extracted using Trizol reagent as described previously [37] from two randomly selected patient samples, one who consumed isoflavones and one who took the placebo. Then, biotin labeled cDNA synthesis, and hybridization with the membrane was performed according to the manufacturer’s instruction to the gene array of our interest. Signals were detected using the optimized buffers and experimental conditions. Data were corrected for background and normalized to a housekeeping gene.

### Statistical Analysis

The sample size was determined from a previous pilot study using the same supplement formulation [22]. Data from the pilot study indicated that total serum estrogen reductions would be 0 and 47 in the control and treatment arm respectively, with a common standard deviation of 36. From the same pilot study we predicted that serum testosterone reduction would be 0 and 1.36 for the control and treatment arm respectively, with a common standard deviation of 2. The sample size was calculated with two primary endpoints, total serum estrogen and testosterone, at a two-sided level of significance of 0.025. Using a two-sample t-test, 43 subjects per group gave us over 95% power to detect the expected difference in estrogen and 80% power to detect the expected difference in testosterone.

Baseline characteristics were compared with t-tests for continuous variables after checking normality assumptions and Fisher’s Exact test for categorical variables. This experiment used a repeated measures design and the analyses for the serum outcomes were conducted using Proc Mixed that allows modeling of both serum outcome means and the covariance structure, which is necessary for these designs. Two separate models were considered, an analysis of covariance model was used to compare groups adjusted for the baseline value of the outcome using an additional variable for the baseline observation and a time class variable for the remaining two time points, and a second model that did not split out the baseline observation as a separate variable. For all serum outcomes and models, a treatment by week interaction was considered. Both models used an autoregressive AR(1) covariance structure which was selected based on model fit statistics from Proc Mixed and the expected correlation structure for the experiment. A level of significance of *p*<0.05 was considered statistically significant. All analyses were performed using SAS version 9.3 TS Level T1M0 (SAS Institute, Inc., Cary, NC).

## Supporting Information

Checklist S1 **CONSORT Checklist.**
(DOC)

Protocol S1 **Trial Protocol.**
(DOC)
