# Supplementary material for: Short-Term Soy Isoflavone Intervention in Patients with Localized Prostate Cancer: A Randomized, Double-Blind, Placebo-Controlled Trial
Source: PLoS One. 2013 Jul 12;8(7):e68331. doi: 10.1371/journal.pone.0068331 (PMC3710024; doi:10.1371/journal.pone.0068331)
Supplement: Protocol S1 — Trial Protocol. (DOC) [file pone.0068331.s002.doc]

**Protocol S1. Role of Soy Isoflavones in Prostate Cancer Prevention: Clinical Protocol**

**Principle Investigators:** Peter Van Veldhuizen, M.D.

**Co-Investigators:**  Jeffrey Holzbeierlein, M.D.

  J. Brantley Thrasher, M.D.

Suman Kambhampati, M.D.

**Study Coordinator:** Sarah Spencer 816-861-4700 Ext. 57665

Kimberley Gomes 816-861-4700 Ext. 57133

Contact Information:

Peter J. Van Veldhuizen, M.D.

Veterans Affairs Medical Center

4801 Linwood Blvd.

Kansas City, MO 64128

Telephone #: (816)861-4700 Ext. 56775

Fax #: (816)922-3323

TABLE OF CONTENTS

1. OBJECTIVES 3-4
2. INTRODUCTION 4-6
3. STUDY PLAN 7-8
4. PATIENT INCLUSION/EXCLUSION CRITERIA 8
5. STUDY CALENDAR 9
6. EFFICACY AND SAFETY MEASUREMENTS 9
7. TISSUE AND SERUM COLLECTION 10-11
8. PATIENT ACCRUAL 11
9. STATISTICAL PLAN 12
10. CRITERIA FOR REMOVAL OF PATIENTS FROM STUDY 13
11. HUMAN SUBJECTS PROTECTION 13-14
12. REFERENCES 15-18

Appendix 1: Informed Consent

Appendix 2: NCI Common Terminology Criteria for Adverse Events (Version 3.0)

1. OBJECTIVES

Commercial soy products have been widely promoted and are consumed by a significant number of patients, either in an effort to prevent or treat prostate cancer. The mechanism of action of these products, appropriate dose, degree of benefit and safety profile, however, remains unknown.^1^ Our central hypothesis is that soy supplements do have beneficial effects in decreasing prostate cancer proliferation and development and, that this effect is related to the phytoestrogenic activity of the soy isoflavones. We propose to examine the effect of a commercially available isoflavone containing soy-based product in patients with adenocarcinoma of the prostate. The patient population to be studied is patients who are scheduled to undergo a radical prostatectomy as the primary treatment for their prostate cancer. This patient population is chosen because the soy isoflavones are more likely to have a benefit early in prostate cancer development and the availability of tissue from prostatectomy specimens is ideal for study the soy effects. The proposed project is a randomized two-arm study comparing a commercial soy supplement to placebo. Specific aim 1 concerns the clinical protocol and specific aims 2&3 the basic science studies.

Specific Aim 1: To assess the effect of soy supplementation on endogenous hormone levels and serum prostate specific antigen. Our hypothesis is that soy supplementation and genistein will decrease serum estrogen and androgen levels, and that these changes will correlate with the molecular effects evaluated in specific aims #2 and #3. Serum levels of testosterone, dihydrotestosterone (DHT), total estrogen, estradiol, sex hormone binding globulin (SHBG), and prostate specific antigen (PSA) will be measured in all groups at the time of randomization (prior to soy supplementation), after two weeks of supplementation and prior to radical prostatectomy (completion of soy supplementation). Serum levels of genistein, insulin-like growth factor-1 (IGF-1) and insulin-like growth factor binding protein-3 (IGFBP-3) will be measured at randomization and after two weeks of supplementation.

**Specific Aim 2: To assess the impact of soy supplementation on the estrogen receptor (ER) including ER alpha and ER beta.** **Our hypothesis is that soy isoflavones decrease prostate cancer proliferation by exerting antiestrogenic effects on ER-α dependant mechanisms, with a resultant down regulation of ER-α expression.** Proposed experiments will examine the direct effect of soy isoflavones on estrogen receptor expression. This effect will be compared with the resultant changes in tumor cell apoptosis and proliferation. Specific experimental approaches include: (i) measurement of ER-α and ER-ß expression in needle biopsy specimens (pre-soy) and from the radical prostatectomy specimens (post-soy), (ii) measurement of apoptotic cell death using the TUNEL assay and (iii) measurement of proliferation indices using PCNA immunohistochemical analyses. Receptor expression levels and degree of apoptosis will be compared between the two groups and will be correlated with patient specific profiles that will include pretreatment tumor stage, PSA, and Gleason’s score.

**Specific Aim 3: To determine the impact of soy isoflavones on cell cycle regulation and associated gene expression. Our hypothesis is that soy isoflavone modulation of the estrogen receptor results in signals that eventually deregulate cell cycle regulatory proteins in the target cells, resulting in the inhibition of tumor proliferation and apoptotic cell death.**  Proposed studies will evaluate effects of soy isoflavones on the gene expression in both the primary prostate tumor and the adjacent non-malignant gland. Specifically, we will: (i) measure cell division cycle-2 kinase (cdc-2) and phosphorylated cdc-2, (ii) evaluate the status of genes essential for cell cycle regulation using cDNA microarray analyses and (iii) further characterize changes in gene expression using Northern and Western Blots. Fresh tumor samples and an area of adjacent normal gland will be obtained on randomized patients. These results will be correlated with the serum hormonal changes identified in specific aim 1 and the estrogen receptor level expression changes identified in specific aim 2.

1. **INTRODUCTION**

Prostate cancer is the most common malignancy in US males and is the second leading cause of malignancy related deaths. In the US, the incidence of this disease continues to increase as our population ages. It is estimated that 230,110 new cases will be diagnosed and 29,900 deaths will occur secondary to prostate cancer in 2004.^2^ Although prostate cancer has been predominantly considered to be a disease of older men, it can also occur in younger men. Sakr et al. (1994) demonstrated high-grade prostatic intraepithelial neoplasia (PIN) in 5% of men in their 4th decade of life, 10% of men in their 5th decade of life and 63% of men in their 7th decade of life. The corresponding figures for invasive carcinoma were 29%, 32% and 64% respectively.^3^ The incidence of low grade PIN was 9% in men who were in only their third decade of life.^4^ Clearly, factors that influence the development of PIN and the subsequent development of an invasive cancer occur early in life. Although the first premalignant changes occur at a young age, the majority of clinical cancers are not detected in most patients until after age 60. This suggests that factors such as a lifetime exposure to a specific diet and/or specific serum androgen levels may not only be important to the ultimate promotion of tumor development but also ideal targets for tumor prevention.^5,6^

The preventive and the therapeutic effect of diet and nutrition on cancer including breast, colon and prostate has received significant attention.^7,8^ As many as one-third of all cancer deaths in the United States can be linked to diet. Significant epidemiological evidence suggests a role of a variety of dietary factors including the degree of lycopene intake, consumption of red meats and the amount of dietary soy.^9-11^ In Asian countries where the average diet consists of 10 times the amount of soy products consumed compared to the average American diet there is a significantly lower incidence of prostate and breast cancer. Further supporting a dietary and environmental influence is the finding that Asian immigrants who have moved to the United States and adopted a Western diet have a prostate cancer incidence that approaches U.S. born males.^12^ These findings have been supported by other studies^13^ and, again, suggest that environmental factors such as dietary soy intake may be responsible for these differences in geographic incidence. Despite the epidemiological data, the specific dietary factors effective in cancer prevention and the mechanism by which specific dietary interventions influence cancer development remains unknown. One potential hypothesis that will be explored in this proposal is that dietary micronutrients may decrease serum testosterone or dihydrotestosterone levels, decreasing the risk of developing prostate cancer. Supporting this premise is epidemiologic data suggesting a higher lifetime exposure to serum androgens leads to an increased risk of developing prostate carcinoma.^14,15^

The isoflavones, which are classified as phytoestrogens, are considered the responsible agents for the anticancer benefit of soy. Asian isoflavone intake is approximately 50-80 mg/day compared to the American intake of 2 to 3 mg/day.^16^ Genistein is felt to be the most active anti-carcinogen agent in soy and it has been targeted as a potential chemopreventative agent.^17,18^ *In vitro* studies using prostate cancer cell lines exposed to genistein have shown a growth inhibiting effect. In a cDNA microanalysis of the LNCaP prostate cancer cell line, the expression of the apoptosis inhibitor, survivin, the cell division cycle kinase-6 (cdc-6) and mitogen-activated protein kinase-6 (MAPK-6) was downregulated when exposed to genistein with a resultant decrease in tumor cell proliferation.^19^ In a second study, genistein induced a G1 cell cycle arrest in a dose-dependant manner.^20^ Although they have not been studied as extensively, other soy isoflavones such as daidzein, equol and biochanin A, modulate the growth of prostate cancer cell lines and also appear to be important components of the beneficial effect of the soy diet.^21,22^ Experiments using animal model systems similarly demonstrate that soy isoflavones prevent or limit the growth and metastases of prostate tumors with a correlative decrease in serum androgen levels.^23-25^

The isoflavones are heterocyclic phenols with close structural homology to estrogens. Individually, isoflavones have weak estrogenic activity, possessing between 1/1,000 and 1/100,000 the activity of synthetic estrogens.^8^ In breast cancer where the role of ERs is better understood, compounds with weak estrogenic activity (e.g. tamoxifen) have an antiestrogenic effect and result in tumor regression.^26^ In prostate cancer, treatment with an agent with estrogenic activity (e.g. diethylstilbestrol/DES) also results in tumor regression in the majority of hormonally sensitive prostate cancers. This response correlates with a decrease in serum androgen levels indicating the primary mechanism of action is the result of androgen ablation. DES, however, exhibits antitumor effects in greater than 50% of patients who have failed initial androgen abalation therapy^27^ suggesting that antitumor mechanisms other than androgen suppression, such as modulation of the estrogen receptor, may be important. The clinical impact and role of estrogen receptor expression or it’s modulation in human prostate cancer has not been defined and will be evaluated in this proposal.^28-31^

Two studies have evaluated the effects of dietary genistein on estrogen receptor expression in the rat prostate. In the first study, animals receiving a continuous diet of genistein demonstrated down regulation of ERβ but not ERα.^32^ A second study noted significant dose-dependent down regulation of ERα, ERβ and the androgen receptor (AR) in male Sprague-Dawley rats receiving dietary genistein.^33^ An additional study examining dihydrotestosterone’s (DHT) induction of prostate androgen-regulated transcript-1 (PART-1) gene expression demonstrated that both genistein and daidzein inhibited DHT–induced expression in a dose dependant manner, offering additional support to a hormonal effect of soy supplementation.^34^

In an experiment in which Lobund-Wistar rats were fed differing concentrations of genistein rich diets, there was inhibition of the expression of both EGF (epidermal growth factor) and the ErB2/Neu receptor in a dose-dependent manner.^35^ In both the androgen-dependent cell line (LNCaP) and androgen-independent prostate cancer cell line (PC-3), genistein inhibited tumor growth in both cell lines, down regulated the cell cycle regulating protein, cyclin B, and upregulated the growth inhibitory protein p21.^36^ This effect in the androgen independent PC-3 cell line again suggests that androgen responsiveness it is not required for growth inhibition by genistein and other mechanisms of action may be important.

There is only limited data regarding the *in vivo* effect of soy isoflavones on human prostate tissue. In a small pilot study that compared resected prostate cancer tissue in a treated versus an untreated control group, there was a trend toward decreased proliferation, as measured by Mib-1 assay, and increased apoptosis, as measured by the TUNEL assay.^37^ In a prospective study of 12,395 Californian Seventh-Day Adventists men, frequent consumption of soymilk was found to result in a 70% reduction in the risk of developing prostate cancer.^38^ In a separate randomized study evaluating the effect of soymilk on serum estrogen and androgen concentration, there was a significant decrease in serum estrone levels but not serum androgen levels.^39^ There are no prior studies on the *in vivo* effect of these hormonal changes on estrogen receptor expression or cell cycle regulation. There is also limited data on the effect of soy isoflavones on non-malignant human prostate tissue. One study has evaluated the effect of genistein on a benign prostatic hypertrophy histoculture and demonstrated decreased cell proliferation as measured by thymidine incorporation.^40^

Serum prostate specific antigen (PSA) remains the most commonly used marker for prostate cancer screening and evaluation of disease recurrence. *In vitro* experiments have demonstrated a decreased in PSA production in response to soy isoflavones.^41,42^ In a small clinical trial, however, 34 patients with an elevated PSA received a soy beverage for a total of 12 weeks with no significant effect on serum PSA levels observed.^43^ The small sample size and the lack of a control arm, though, limited the study. A recently published study evaluated the effects of a genistein-rich commercial supplement on serum PSA in 62 men with a histologically confirmed prostate carcinoma. The trial included patients with recurrence after prostatectomy (RRP)(9), after radiation therapy (17), after both radiation and RRP (6), after hormonal therapy (14), and patients receiving surveillance only (16). Of the 52 evaluable patients who had received at least six months of supplementation, one patient had PSA reduction of >50% and 8 had PSA responses that were <50%. PSA responses were seen preferentially in patients only receiving active surveillance with 8 of 13 patients having either a stable or decreasing level.^44^ The authors postulate that prior treatment may have affected genistein’s ability to concentrate in the prostate gland, thereby decreasing the therapeutic effect. Our proposed randomized study, which will evaluate a group of previously untreated patients, will further delineate the soy isoflavone effect on PSA levels, evaluate the dose relationship, and correlate these changes with the molecular effects on both normal and malignant prostate tissue.

There has been no significant toxicity reported with comparable soy supplementation doses in other studies and no toxicity was seen in a pilot trial performed at our institution.^45,46^ Soy supplementation has been demonstrated to decrease endogenous hormone levels such as free T3, DHEA and serum cortisol.^47-49^ Longer-term supplementation could lead to toxicity as a result of the chronic effects on these hormone levels but is unlikely in this setting of short-term supplementation. Potential estrogenic effects include an increase risk of thrombosis, gynecomastia, fluid retention and, rarely, elevation of liver function tests.

**Significance and Relevance to Veterans Heath:** In the year 2004, prostate cancer will account for approximately one third of the new cases of cancer diagnosed in men in the United States, including our veteran population. Chemopreventive strategies that can reduce the tremendous cost, both economically and in terms of morbidity/mortality, of this disease are desperately needed. Although there is preclinical and epidemiological data suggesting a benefit of the soy isoflavones and a soy-based diet, there is limited information regarding the *in vivo* effects of soy supplementation on human prostate cancer and benign prostate tissue. These proposed studies would define both the dose effect and the molecular effects of soy isoflavones on human prostate cancer; and further clarify the chemopreventive potential of a soy-based diet.

1. STUDY DESIGN: CLINICAL PROTOCOL

Rationale:

African-Americans who have higher serum androgen levels also have the highest incidence of prostate cancer. The incidence of prostate cancer is much lower in Asian countries which also correlates both with a decrease in serum androgen levels and a soy-based diet.^5,6^ Prostate cancer is nonexistent in individuals castrate at a young age and in patient populations deficient in 5 alpha-reductase, which is important for the conversion of testosterone to 5-DHT.^50,51^ There is also a decreased incidence in patients with chronic disorders that are associated with lower levels of serum androgens such as diabetes mellitus and cirrhosis.^52-54^ These findings suggest that an agent that can consistently reduce baseline serum androgens level without significant side effect, could have a significant impact in prostate cancer chemoprevention. There have been variable reports with regard to the effect of soy on serum testosterone levels with two studies demonstrating no effect with the ingestion of soy milk. ^39,55^ The data from our pilot trial, though, demonstrated a trend toward decreasing testosterone levels. Since we are using a different soy supplement and a change in testosterone levels could contribute to the molecular effects seen, co-analysis of serum androgen levels in addition to serum estrogen levels is required. The evaluation of IGF-1 is important as epidemiologic studies have suggested that high circulating IGF-1 levels are associated with an increased risk of prostate cancer and this increase is associated with dietary factors. The effect of soy isoflavones on serum IGF-1 and IGFBP-3 has not previously been evaluated in this setting.^56,57^

Study Design:

Histologically Confirmed Adenocarcinoma of the Prostate

Scheduled to Undergo Prostatectomy for Primary Curative Therapy

Randomize

Placebo Soy Supplement

# Experimental Design:

# This will be a two-arm randomized study comparing a placebo control arm to supplementation with a commercial soy supplement. The time of initial biopsy to the time the surgery scheduled is typically 2-4 weeks in normal setting. Patients will be treated for a minimum of two weeks and continue treatment until the time of their prostatectomy. A total of 86 patients will be randomized (43 patients/arm).

Supplementation with Soy Isoflavones:

Dose Levels:

Dose 0: No soy (Placebo).

Dose 1: Four Capsules Twice Daily (240 mg of total isoflavones).

Treatment: The commercial soy supplement to be used is *Flav-Ein* (3B’s LTD, Lexena, Kansas)*.* This product was chosen because it is a pure soy extract and the individual concentration of each soy isoflavone is closely equivalent to a soy-based diet. It is supplied in capsular form. Each capsule contains 560 mg of pure soybean extract and 100mg of a non-soy legume (see appendix). Each capsule contains approximately 30mg of total isoflavones. The placebo will consist of an equivalent capsule with only the 100mg legume additive and no soy supplement.

1. ELIGIBILITY/EXCLUSION CRITERIA:

All patients must meet the following eligibility and exclusion criteria:

1. Histologically proven adenocarcinoma of the prostate.
2. Surgical candidate for radical prostatectomy.
3. During time of study period, patients must agree not to take any new vitamin supplementation or herbal remedy.
4. Must not be receiving concurrent chemotherapy, radiation or hormonal therapy.
5. No history of prior allergy and intolerability to soy-based products.
6. Must not have been taking any soy supplementation or soy isoflavones within 90 days prior to study enrollment.
7. Must be able to safely be on replacement for period of at least two weeks prior to the scheduled prostatectomy.
8. All patients must have been informed of the investigational natures of this study and must sign and give written informed consent in accordance with institutional and federal guidelines.
9. **STUDY CALENDAR**

| REQUIRED  STUDIES | Study Enrollment | Two Weeks | Preprostatectomy |
| --- | --- | --- | --- |
|  |  |  |  |
| EXAM |  |  |  |
| History/PE | X | X | X |
| Weight | X | X | X |
| PS | X | X | X |
| Toxicity | X | X | X |
|  |  |  |  |
| SERUM# |  |  |  |
| PSA | X | X | X |
| Estrogen(total) | X | X | X |
| Estradiol | X | X | X |
| Testosterone | X | X | X |
| DHT | X | X | X |
| SHBG | X | X | X |
| IGF-1 | X | X |  |
| IGFBP-3 | X | X |  |
| Genistein* | X | X |  |
|  |  |  |  |
| TREATMENT |  |  |  |
| Soy Isoflavone | X | X | X |

# Serum from a 15 ml red top tube to be collected and stored at -70^0^.

*Genistein collection (urine and serum): Serum collection in AM prior to study enrollment (baseline level) and 3 hours after capsule ingestion at week two (peak level). Urine collections will be first morning specimen.

1. EFFICACY AND SAFETY MEASUREMENTS

Informed Consent/Adverse Events:

Informed consent will be obtained prior to study enrollment for all patients as approved by our institutional IRB/Human subjects committee. All adverse events will be reported to the institutional IRB.

Tumor Response and Assessment:

Tumor response will be assessed by PSA, pathologic and molecular changes. Patient profiles will be developed which include tumor stage, Gleason’s score and pretreatment PSA.

Toxicity Assessment:

Patients will be evaluated for toxicity at week two, just prior to prostatectomy and postoperatively. Toxicity will be recorded using version 3.0 of the NCI common toxicity criteria (website: [http://ctep.cancer.gov](http://ctep.cancer.gov/)). Please see human subjects section (page 64) for further information on reporting serious adverse events.

Dietary Assessment:

A diet history will be obtained at baseline and prior to surgery characterize the subject's usual dietary soy intake and to assess for any changes during the course of the study treatment. The diet history will be conducted by the clinical research nurse and will include a 24-hour dietary recall, usual eating habits, food intolerances, preferences, and a validated soy food frequency questionnaire.^58^

1. TISSUE AND SERUM COLLECTION

Laboratory Collection and Assessment:

PSA, serum testosterone, 5-DHT, total estrogen and estradiol levels will be assessed at baseline, at two weeks and prior to prostatectomy (Table I). These will be done using standardized assays at the KCVA clinical laboratory. Serum levels of IGF-1, IGFBP-3 and genistein will be drawn at baseline after two weeks of supplementation. Genistein levels will be measured in Dr. Reed’s laboratory by the method described below. IGF-1 and IGFBP-3 will be completed in Dr. Banerjee’s laboratory using a standard RIA assay (ALPCO Diagnostics). All samples will be drawn in the early AM and in the fasting state.

Tissue Collection:

For the immunostaining(ER-α, ER-β, apoptosis, proliferation, cdc-2, pcdc-2) and in situ hybridization studies(ER-α, ER-β , cdc-2, pcdc-2) 10 paraffin embedded slides from the diagnostic biopsy (pre-soy) and 10 paraffin embedded slides from the prostatectomy specimen (post-soy), will be obtained on all enrolled patients. These slides will be obtained after pathologic processing and, if needed, additional paraffin sections will be available. In addition, at prostatectomy and prior to pathologic processing, the urological surgeon will obtain core samples of both adjacent normal prostate and tumor from each specimen. This tissue is collected in the operating room suite. Once collected, the samples are immediately snap frozen and stored in liquid nitrogen until experimental analysis. An H&E stained section will be obtained from each core to ensure the presence of tumor or adjacent normal tissue. This method of tissue collection has previously been reported^59^ is currently being successfully utilized at the Kansas City VAMC.

Genistein Levels and Specific Methods:

Serum genistein levels will be measured at baseline (time of study enrollment) and at the week two assessment. Genistein levels will be drawn three hours after the AM dose at the week two visit. In addition, urine genistein levels will be analyzed at baseline and at the two-week visit. Both urine samples will be first morning urine specimens. Samples will be collected and stored at –70^0^C. Samples will be analyzed in groups of 24-36, correlating with the accrual of every 12-18 patients. Prior studies have demonstrated a genistein half-life of in the range of 3-6 hours with peak serum concentration occurring of approximately 3 hour following a supplementation dose.^45,60^ In addition, prior urine sampling indicates that most of the genistein excreted is in the 12-24 hour fraction after dosing.

The measurement of genistein in aliquots of plasma (1.0 ml) or urine (5.0 ml) will be performed as described previously.^61^ For measurement of free genistein, plasma or urine specimens will be loaded on Extrelut™ QE columns (EM Scientific), washed with water, and eluted with ethyl acetate. Analysis of total genistein (free and conjugated) will be performed by incubation of the plasma and urine samples with β-glucuronidase and aryl sulfatase for 18 hrs at 37° C prior to solid phase extraction. Eluent will be evaporated to dryness under reduced pressure and the residue dissolved in mobile phase for analysis by HPLC. Analysis will use a YMC ODS-AM reverse phase HPLC column (5 µ, 4.6 x 250 mm) eluted with a gradient of 2 mM acetic acid in water and methanol. Eluent will be monitored by absorbance at 260 nm. Authentic genistein (Sigma-Aldrich) will be used as an analytical standard. This procedure has been successfully used for the analysis of genistein and other isoflavones in the urine and plasma of human subjects consuming soymilk.^60^ Serum and urine genistein levels in the placebo group will depend on the baseline dietary isoflavone intake but detectable levels can be measured in subjects at intake levels typical for the Western diet.^62^

#### Tissue and Serum Banking:

#### All patients will have a 15 ml red top drawn at enrollment, at two weeks and at the completion of soy supplementation. From this, we will use a 1ml aliquot for genistein analysis and a 1ml aliquot IGF-1/IGFBP-3 analysis. The remaining sample will be labeled and stored in –70^0^C. Our studies will also require one tissue core of tumor and adjacent normal tissue. When feasible, at least one additional core of both tumor and normal tissue will be collected according to the tissue collection methods listed above. Samples will then be labeled and stored in liquid nitrogen. These samples will be available for additional investigation including potential future collaborative efforts. The KCVA is also one of the sites selected for the planned VA tissue and biorepository project and these samples could readily be contributed to that tissue bank.

####

1. PATIENT ACCRUAL

Patients will be accrued at the Kansas City Veterans Affairs medical center (KCVA). The Kansas City VAMC is the tertiary referral center for the western orbit of VISN 15 and serves a large veteran population. Patients from the Leavenworth and Topeka VAMC’s who desire prostatectomy for treatment of their prostate cancer are also referred to the Kansas City VA’s urologic surgeons, Dr. Thrasher and Dr. Holzbeierlein. Each year 100-120 new cases of prostate cancer are diagnosed, the majority of which are early stage, with an average 40-50 prostatectomies performed yearly. The majority of the remaining patients are treated with either external beam radiation therapy, brachytherapy or hormonal therapy. In the pilot study that was performed at the KCVA alone, 13 patients were enrolled in less than six months, with nine of the patients being enrolled in a three-month time period. Patient acceptance approached 100%, given the low toxicity and ease of administration of the treatment intervention.

### STATISTICAL PLAN:

The primary objective of this study is to evaluate the molecular effects of soy isoflavones on human prostate cancer as well as the effects on serum androgen and estrogen levels. The goal is to randomize a total of 86 patients (43 to the control/placebo arm and 43 to the treatment/240mg soy arm). We will use a placebo and the lab personnel as well as clinicians, nurses and study coordinators will be blinded to treatment arm.

Randomization

Patients will be sequentially randomized into one of the two arms. Random permuted block randomization will be utilized. An independent statistician will generate the codes and will provide a copy of them to the VA research pharmacy. The study coordinator will notify the research pharmacy of the subjects ID number and the pharmacy will prepare the medication and distribute it to the patient.

Data Management

Two versions of an Access database will be created that mimics the patient data collection forms. One will be called primary and the other verification. Data will be entered into each database and then compares will be run to ensure the number of patients in each database is identical and that each data item is identical. This will be done each time data is to be entered. Logic, edit and range checks will be performed after compares have shown that the data in the primary and verification databases are identical. After the final patients data have been entered, compared and all logic, edit and range checks have been complete the database will be locked and data analysis will begin.

Sample Size and Power

Two primary endpoints for specific aim 1 are total serum estrogen and testosterone. The goal is to determine if there is a difference in the change in these values between the two groups. Each of these endpoints will be tested at the 0.025 level of significance. Data from the pilot study indicated that total serum estrogen reductions will be 0 and 47 in the control and treatment arm respectively, with a common standard deviation of 36. From the same pilot study we expect that serum testosterone reduction will be 0 and 1.36 for the control and treatment arm respectively, with a common standard deviation of 2. Given this information, we want a sufficient sample size to have at least 80% power to detect these changes for both endpoints at the 0.025 level of significance. Using a two-sample t-test, 43 subjects per group will give us over 95% power to detect the expected difference in estrogen and 80% power to detect the expected difference in testosterone.

Statistical Analysis Plan:

Categorical demographic variables and baseline characteristics will be summarized by frequencies and percentages, overall and by group. Quantitative demographic variables and baseline characteristics will be summarized by means and standard deviations, overall and by group. For specific aim 1, the primary endpoints are total estrogen and testosterone. We will summarize baseline, post-treatment and change for these variables by means and standard deviations for both groups. We will then compare the change in each of these endpoints using a two-sample t-test, at the 0.025 level of significance. Similar procedures will be done on the other serum endpoints; estradiol, PSA, 5-DHT, IGF-1, and IGFBP-3, however, these tests will be at the 0.05 level of significance.

In an exploratory manner, we will use general linear models to determine if some or any of the baseline characteristics are predictive of change in these variables while controlling for treatment. We will also use Pearson’s correlation to determine if changes in the biomarkers are correlated with one another. For specific aim 2, the primary endpoints are ER-α and ER-β. Similar analyses to what was used for specific aim 1 will be applied to these endpoints as well at the 0.05 level of significance.

1. CRITERIA FOR REMOVAL FROM STUDY
2. Progression of disease.
3. Resultant delay of surgery treatment greater than 3 weeks.
4. Unacceptable toxicity: there will be no dose reductions, any patient with unacceptable toxicity will be removed and proceed with planned prostatectomy.
5. The patient may withdraw from the study at any time for any reason.

1. HUMAN SUBJECTS:
2. **Risk of Subjects**

Human Subject Involvement and Characteristics: This study will involve patients who are scheduled to undergo radical prostatectomy for treatment of their prostate cancer. Patient will receive variable doses of a soy supplement from the time of study enrollment until the time of their planned surgery. All patients must have histologic confirmation of adenocarcinoma of the prostate must be surgical candidates as dictated by their disease stage and health status. Because of disease being studied, all patients will be males of all ethnic backgrounds who are greater than 18 years of age with the expected age range of 55-70. All patients must meet the following additional eligibility and exclusion criteria: a) patients must agree not to take any new vitamin supplementation or herbal remedy, b) must not be receiving concurrent chemotherapy, radiation or hormonal therapy, c) no history of prior allergy and intolerability to soy-based products, d) must not have been taking any soy supplementation or soy isoflavones within 90 days prior to study enrollment, e) must be able to safely be on supplements for period of at least two weeks prior to the scheduled prostatectomy, f) all patients must have been informed of the investigational natures of this study and must sign and give written informed consent in accordance with institutional and federal guidelines. A total of 86 patient will be accrued over a three-year period.

Sources of Materials: Sources of research material include serum, tissue specimens and clinical data which will include toxicity data and demographic characteristics. Serum will be collected at study enrollment, after two weeks, and at the time of prostatectomy. Prostate tissue will be collected at the time of the diagnostic biopsy and prostatectomy. All material will be collected specifically for research purposes.

Potential Risks: Although there was no toxicity seen in the pilot trial, the potential research risk of treating with an estrogenic agent includes an increase risk of thrombosis, gynecomastia, fluid retention and elevation of liver function tests. Based on preliminary data and prior studies, these risks are of low likelihood and readily reversible with the exception of thrombosis. Although it is of low likelihood, thrombosis can lead to fatal pulmonary embolism in a small percentage of case. Phlebotomy risks include pain at the injection site and infection. There are no known social, psychological and legal risks. Therapeutic risks include postoperative thrombosis, infection, impotency and incontinence which all can occur with a standard prostatectomy**.** The thrombotic risk is expected to be extremely with no risk reported in other published trials of soy supplementations.

1. **Adequacy of Protection for Risks**

Recruitment and Informed Consent: Potential patients will subjects who have been referred to the urology clinics at the VAMC for evaluation of a potential prostate cancer (elevated PSA, DRE) and have subsequent documentation of an invasive prostate cancer. Only patients who are surgical candidates and have chosen surgical as their treatment choice will be offered participation. Patient will be provided consent form, information on soy supplementation and study aims in lay language. Potential candidates will be identified by participating investigators (Dr. Van Veldhuizen, Dr. Thrasher, Dr. Holzbeierlein) who will also answer questions and provide additional education on the study. Patients will be allowed to read and take the consent home. Final consent will be obtained by Tracina Renaud (Research Associate). Copy of the informed consent will be submitted to the IRB and maintained in the patient record. The IRB the Kansas City VAMC has not authorized a modification or waiver of the elements of consent or its documentation. All clinical personal have had human subjects/informed consent training.

Protection Against Risk: All adverse effects will be reported to the institutional IRB and all toxicity will be graded according to the NCI Common Toxicity Criteria, Version 3.0(**http://ctep.cancer.gov)**. **{**The University of Kansas Data Monitoring safety board(DMSB) will oversee this trial. Their review includes data quality and timeliness, adherence to study protocol, informed consent procedures and adverse events. Any grade 3 or 4 adverse effect (SAE) will result in immediate discontinuation of soy supplement and removing from treatment protocol. SAE’s will be immediately recorded and reported to the KCVA human subjects committee and the DMSB.**}** If any thrombosis occurs, patients will immediately be removed from study and will be treated promptly using standard anticoagulation protocols. Interim analysis on toxicity data will be performed every six months to ensure no differences in risks are seen with the treatment compared to the control arm. Every effort will be made to protect patient confidentiality including; a) all data will be keep in a secured area, and b) patient information will be coded with the names of the patients kept in a locked file.

1. **Potential Benefit of the Proposed Research to the Subject and Others**

Risk/Benefit Ratio: Although the direct benefit to the participating such is low, there is a substantial potential benefit to society if this study can aid in identifying a potential approach to prostate cancer prevention. The risk and toxicities of this intervention are also expected to be very minimal.

1. **Importance of Knowledge to be gained**

Prostate cancer is an enormous health problem with over 200,000 new cases diagnosed and 30,000 deaths occurring each year in the US alone. If a preventive strategy or a new chemopreventive agent could prevent only 5% of prostate cancers, 10,000 patients each year would not have to suffer from this disease. The risks of the treatment intervention in this study are very low compared to the potential knowledge gained.

1. LITERATURE CITED
2. Golwyn S, Lazinsky A, Wei H: Promotion of health by soy isoflavones: efficacy, benefit and safety concerns. Drug Metabol Drug Interact 2000;17(1-4): 261-289.
3. Jemal A, Tiwari RC, Murray T, Ghafoor A, Samuels A, Ward E, Feuer EJ, Thun M: Cancer Statistics, 2004. CA Cancer Journal for Clinicians 2004;54:8-29.
4. Sakr WA, Grignon DJ, Crissman JD, Heilbrun LK, Cassin BJ, Pontes JJ, Haas GP: High grade prostatic intraepithelial neoplasia (HGPIN) and prostatic adenocarcinoma between the ages of 20-69: an autopsy study of 249 cases. In Vivo 1994;8(3):439-443.
5. Sakr WA, Haas GP, Cassin BF, Pontes JE, Crissman JD: The frequency of carcinoma and intraepithelial neoplasia of the prostate in young male patients. J Urol 1993;150:379-385.
6. Debes JD, Tindall DJ: The role of androgens and the androgen receptor in prostate cancer. Cancer Lett 2002;10;187(1-2):1-7.
7. Adlercreutz H, Mazur W: Phyto-oestrogens and Western Diseases. Annals of Medicine 1997;29:95-120.
8. Shirai T, Asamoto M, Takahashi S, Imaida K: Diet and prostate cancer. Toxicology 2002;181-182:89-94.
9. Cassidy A: Potential risks and benefits of phytoestrogen-rich diets. Int J Vitam Nutr Res 2003;73(2):120-126.
10. Hadley CW, Miller EC, Schwartz SJ, Clinton SK: Tomatoes, lycopene, and prostate cancer: progress and promise. Exp Biol Med (Maywood) 2002;227(10):869-880.
11. Ferguson LR: Meat consumption, cancer risk and population groups within New Zealand. Mutat Res 2002;506-507:215-224.
12. Messina MJ: Legumes and soybeans: overview of their nutritional profiles and health effects. Am J Clin Nutr 1999;70(3 Suppl):439S-450S.
13. Muir CS, Nectoux J, Staszewski J: The epidemiology of prostate cancer: geographical distribution and time trends. Acta Oncol 1991;30:133-140.
14. Shimizu H, Ross RK, Bernstein, et al: Cancers of the prostate and breast among Japanese and white immigrants in Los Angeles County. Br J Cancer 1991;63:963-966.
15. Moyad MA: Lifestyle/dietary supplement partial androgen suppression and/or estrogen manipulation. A novel PSA reducer and preventive/treatment option for prostate cancer? Urol Clin North Am 2002 Feb; 29(1):115-124.
16. Rao GN: Influence of diet on tumors of hormonal tissues. Prog Clin Biol Res 1996;394:41-56.
17. Mazur W: Phytoestrogen content in foods. Bailliere’s Clinical Endocrinology and Metabolism 1998;12(4):729-742.
18. Lamartiniere CA, Cotroneo MS, Fritz WA, Wang J, Mentor-Marcel R, Elgavish A: Genistein chemoprevention: timing and mechanisms of action in murine mammary and prostate. J Nutr 2002;132(3):552S-558S.
19. Sarkar FH, Li Y: Mechanisms of cancer chemoprevention by soy isoflavone genistein. Cancer Metastasis Rev 2002;21(3-4):265-280.
20. Suzuki K, Koike H, Matsui H, Ono Y, Hasumi M, Nakazato H, Okugi H, Sekine Y, Oki K, Ito K, Yamamoto T, Fukabori Y, Kurokawa K, Yamanaka H: Genistein, a soy isoflavone, induces glutathione peroxidase in the human prostate cancer cell lines LNCaP and PC-3. Int J Cancer 2002;99(6):846-852.
21. Shen JC, Klein RD, Wei Q, Guan Y, Contois JH, Wang TT, Chang S, Hursting SD: Low-dose genistein induces cyclin-dependent kinase inhibitors and G(1) cell-cycle arrest in human prostate cancer cells. Mol Carcinog 2000;29(2):92-102.
22. Rice L, Samedi VG, Medrano TA, Sweeney CA, Baker HV, Stenstrom A, Furman J, Shiverick KT: Mechanisms of the growth inhibitory effects of the isoflavonoid biochanin A on LNCaP cells and xenografts. Prostate 2002;52(3):201-12.
23. Hedlund TE, Johannes WU, Miller GJ: Soy isoflavonoid equol modulates the growth of benign and malignant prostatic epithelial cells in vitro. Prostate 2003;54(1):68-78.
24. Pollard M, Wolter W, Sun L: Prevention of induced prostate-related cancer by soy protein isolate/isoflavone-supplemented diet in Lobund-Wistar rats. In Vivo 2000;14(3):389-392.
25. Aronson WJ, Tymchuck CN, Elashoff RM, McBride WH, McLean C, Wang H, Heber D: Decreased growth of human prostate LNCaP tumors in SCID mice fed a low-fat, soy protein diet with isoflavones. Nutr Cancer 1999;35(2):130-136.
26. Zhou JR, Yu L, Zhong Y, Nassr RL, Franke AA, Gaston SM, Blackburn GL: Inhibition of orthotopic growth and metastasis of androgen-sensitive human prostate tumors in mice by bioactive soybean components. Prostate 2002;53(2):143-153.
27. Campos SM, Winer EP: Hormonal therapy in postmenopausal women with breast cancer. Oncology 2003;64(4):289-299.
28. Malkowicz SB: The role of diethylstilbestrol in the treatment of prostate cancer. Urology 2001;58(2 Suppl 1):108-113.
29. Tsurusaki T, Aoki D, Kanetake H, Inoue S, Muramatsu M, Hishikawa Y, Koji T: Zone-dependent expression of estrogen receptors alpha and beta in human benign prostatic hyperplasia. J Clin Endocrinol Metab 2003;88(3):1333-1340.
30. Linja MJ, Savinainen KJ, Tammela TL, Isola JJ, Visakorpi T: Expression of ERalpha and ERbeta in prostate cancer. Prostate 2003;55(3):180-186.
31. Signoretti S, Loda M: Estrogen receptor beta in prostate cancer: brake pedal or accelerator? Am J Pathol 2001;159(1):13-16.
32. Cunha GR, Wang YZ, Hayward SW, Risbridger GP: Estrogenic effects on prostatic differentiation and carcinogenesis. Reprod Fertil Dev 2001;13(4):285-296.
33. Dalu A, Blaydes BS, Bryant CW, Latendresse JR, Weis CC, Barry Delclos K: Estrogen receptor expression in the prostate of rats treated with dietary genistein. J Chromatogr B Analyt Technol Biomed Life Sci 2002;777(1-2):249-260. .
34. Fritz WA, Wang J, Eltoum IE, Lamartiniere CA: Dietary genistein down-regulates androgen and estrogen receptor expression in the rat prostate. Mol Cell Endocrinol 2002;186(1):89-99.
35. Yu L, Blackburn GL, Zhou JR: Genistein and daidzein downregulate prostate androgen-regulated transcript-1 (PART-1) gene expression induced by dihydrotestosterone in human prostate LNCaP cancer cells. J Nutr 2003;133(2):389-392.
36. Dalu A, Haskell JF, Coward L: Genistein, a component of soy, inhibits the expression of the EGF and ErbB2/Neu receptors in the rat dorsolateral prostate. Prostate 1998;37(1):36-43.
37. Davis JN, Singh B, Bhuiyan M, Sarkar FH: Genistein-induced upregulation of p21WAF1, downregulation of cyclin B, and induction of apoptosis in prostate cancer cells. Nutr Cancer 1998;32(3):123-131.
38. Flanigan RC, Shankey TV, Wojcik E, Creech S, Waters WB, Campbell SC, Fairbanks T: The Impact of Soy Isoflavones on prostate cancer proliferation and apotosis: Pilot Data. J of Urol(Abstract) 2003:4,supplement.
39. Jacobsen BK, Knutsen SF, Fraser GE: Does high soymilk intake reduce prostate cancer incidence? The Adventist Health Study (United States). Cancer Causes Control 1998;9(6):553-557.
40. Nagata C, Takatsuka N, Shimisu H, Hayashi H, Akamatsu T, Murase K: Effect of soymilk consumption on serum estrogen and androgen concentration in Japanese men. Cancer Epidemiol Biomarkers Prev 2001; 10(3):170-184.
41. Geller J, Sionit L, Partido C, Li L, Tan X, Youngkin T, Nachtsheim D, Hoffman RM: Genistein inhibits the growth of human-patient BPH and prostate cancer in histoculture. Prostate 1998;34(2):75-79.
42. Rosenberg Zand RS, Jenkins DJ, Brown TJ, Diamandis EP: Flavonoids can block PSA production by breast and prostate cancer cell lines. Clin Chim Acta 2002;317(1-2):17-26.
43. Davis JN, Kucuk O, Sarkar FH: Expression of prostate-specific antigen is transcriptionally regulated by genistein in prostate cancer cells. Mol Carcinog 2002;34(2):91-101.
44. Urban D, Irwin W, Kirk M, Markiewicz MA, Myers R, Smith M, Weiss H, Grizzle WE, Barnes S: The effect of isolated soy protein on plasma biomarkers in elderly men with elevated serum prostate specific antigen. J Urol 2001;165(1):294-300.
45. DeVere White RW, Hackman RM, Soares SE, Beckett LA, Li Y, Sun B. Effects of a genistein-rich extract on PSA levels in men with a history of prostate cancer. Urology 2004;63:259-263.
46. Busby MG, Jeffcoat AR, Bloedon LT, et al. Clinical characteristics and pharmacokinetics of purified soy isoflavones: single-dose administration to healthy men. Am J Clin Nutr 2002;75:126-136.
47. Takimoto CH, Glover K, Huang X, et al. Phase I pharmacokinetics and pharmacodynamic analysis of unconjugated soy isoflavones administered to individuals with cancer. Cancer Epidemiol Biomarkers Prev 2003;12:1213-1221.
48. Doerge DR, Sheehan DM: Goitrogenic and estrogenic activity of soy isoflavones. Environ Health Perspect 2002;110 Suppl 3:349-353.
49. Bell DS, Ovalle F: Use of soy protein supplement and resultant need for increased dose of levothyroxine. Endocr Pract 2001;7(3):193-194.
50. Duncan AM, Underhill KE, Xu X, Lavalleur J, Phipps WR, Kurzer MS: Modest hormonal effects of soy isoflavones in postmenopausal women. J Clin Endocrinol Metab 1999;84(10):3479-3484.
51. Hochberg Z, Chayen R, Reiss N, Falik Z, Makler A, Munichor M, Farkas A, Goldfarb H, Ohana N, Hiort O: Clinical, biochemical, and genetic findings in a large pedigree of male and female patients with 5 alpha-reductase 2 deficiency. J Clin Endocrinol Metab 1996;81(8):2821-2827.
52. Geller J, Soinit L: Castration-like effects on the human prostate of a 5 alpha-reductase inhibitor, finasteride. J Cell Biochem Suppl 1992;16H:109-112
53. Weiderpass E, Ye W, Vainio H, Kaaks R, Adami HO: Reduced risk of prostate cancer among patients with diabetes mellitus. Int J Cancer 2002;102(3):258-261.
54. Araki H, Mishina T, Miyakoda K, Fujiwara T, Kobayashi T: An epidemiological survey of prostatic cancer from the Annual of the Pathological Autopsy Cases in Japan. Tohoku J Exp Med 1980;130(2):159-164.
55. Kaymakoglu S, Okten A, Cakaloglu Y, Boztas G, Besisik F, Tascioglu C, Yalcin S: Hypogonadism is not related to the etiology of liver cirrhosis. J Gastroenterol 1995;30(6):745-750.
56. Allen NE, Appleby PN, Davey GK, Key TJ. Soy milk intake in relation to serum sex hormone levels in British men. Nutr Cancer 2001;41(1-2):41-46.
57. Gunnel D, Oliver SE, Peter TJ, et al. Are diet-prostate cancer associations mediated by the IGF axis? A cross-sectional analysis of diet, IGF-1 and IGFBP-3 in healthy middle-aged men. Br J Cancer 2003;88(11):1682-1686.
58. Holmes MD, Pollack MN, Hankinson SE. Lifestyle correlates of plasma insulin-like growth factor 1 and insulin-like growth factor binding protein 3 concentrations. Cancer Epidemiol Biomarkers Prev 2002;11(9):862-867.
59. Frankenfeld CL, Patterson RE, Horner NK, Neuhouser ML, Skor HE, Kalhorn TF, Howald WN, Lampe JW. Validation of a soy food-frequency questionnaire and evaluation of correlates of plasma isoflavone concentrations in postmenopausal women. Am J Clin Nutr 2003 Mar;77(3):674-80.
60. Wheeler TM, Lebovitz RM. Fresh Tissue Harvest for Research From Prostatectomy Specimens. The Prostate 1994;25:274-279.
61. Bloedon LT, Jeffcoat AR, Lopaczynski W, Schell MJ, Black TM, Dix KJ, Thomas BF, Albright C, Busby MG, Crowell JA, Zeisel SH: Safety and pharmacokinetics of purified soy isoflavones: single-dose administration to postmenopausal women. Am J Clin Nutr 2002;76(5):1126-1137.
62. Zhang Y, Wang GJ, Song TT, Murphy PA, Hendrich S: Urinary disposition of the soybean isoflavones daidzein, genistein and glycitein differs among humans with moderate fecal isoflavone degradation activity. J Nutr 129:957-962.
63. Setchell KD, Brown NM, Desai PB, et.al. Bioavailability, disposition, and dose-response effects of soy isoflavones when consumed by healthy women at physiologically typical dietary intakes. J Nutr 2003;133(4):1027-1035.
